# Toll like receptor-3 ligand poly-ICLC promotes the efficacy of peripheral vaccinations with tumor antigen-derived peptide epitopes in murine CNS tumor models

**DOI:** 10.1186/1479-5876-5-10

**Published:** 2007-02-12

**Authors:** Xinmei Zhu, Fumihiko Nishimura, Kotaro Sasaki, Mitsugu Fujita, Jill E Dusak, Junichi Eguchi, Wendy Fellows-Mayle, Walter J Storkus, Paul R Walker, Andres M Salazar, Hideho Okada

**Affiliations:** 1 Department of Neurological Surgery, University of Pittsburgh School of Medicine, Pittsburgh, USA; 2 Department of Surgery, University of Pittsburgh School of Medicine, Pittsburgh, USA; 3 Departments of Dermatology and Immunology, University of Pittsburgh School of Medicine, Pittsburgh, USA; 4 Brain Tumor Program, University of Pittsburgh Cancer Institute, Pittsburgh, USA; 5 Division of Oncology, Geneva University Hospital, Geneva, Switzerland; 6 Oncovir Inc., Washington D.C., USA

## Abstract

**Background:**

Toll-like receptor (TLR)3 ligands serve as natural inducers of pro-inflammatory cytokines capable of promoting Type-1 adaptive immunity, and TLR3 is abundantly expressed by cells within the central nervous system (CNS). To improve the efficacy of vaccine strategies directed against CNS tumors, we evaluated whether administration of a TLR3 ligand, polyinosinic-polycytidylic (poly-IC) stabilized with poly-lysine and carboxymethylcellulose (poly-ICLC) would enhance the anti-CNS tumor effectiveness of tumor peptide-based vaccinations.

**Methods:**

C57BL/6 mice bearing syngeneic CNS GL261 glioma or M05 melanoma received subcutaneous (s.c.) vaccinations with synthetic peptides encoding CTL epitopes- mEphA2 (671–679), hgp100 (25–33) and mTRP-2 (180–188) for GL261, or ovalbumin (OVA: 257–264) for M05. The mice also received intramuscular (i.m.) injections with poly-ICLC.

**Results:**

The combination of subcutaneous (s.c.) peptide-based vaccination and i.m. poly-ICLC administration promoted systemic induction of antigen (Ag)-specific Type-1 CTLs expressing very late activation antigen (VLA)-4, which confers efficient CNS-tumor homing of vaccine-induced CTLs based on experiments with monoclonal antibody (mAb)-mediated blockade of VLA-4. In addition, the combination treatment allowed expression of IFN-γ by CNS tumor-infiltrating CTLs, and improved the survival of tumor bearing mice in the absence of detectable autoimmunity.

**Conclusion:**

These data suggest that poly-ICLC, which has been previously evaluated in clinical trials, can be effectively combined with tumor Ag-specific vaccine strategies, thereby providing a greater index of therapeutic efficacy.

## Background

Our long-term goal is to develop safe and effective immunotherapeutic modalities for CNS tumors, such as gliomas. To this end, we have been directing our major focus on factors that promote the efficacy of peripheral vaccinations against CNS tumor-associated or specific Ags. Indeed, our recent study using adoptive transfer of *ex vivo *activated T-cells has demonstrated that a Type-1 phenotype for tumor-Ag specific CTLs is critical for efficient CNS tumor-tropism and for the resulting anti-tumor therapeutic efficacy, which can be further facilitated by genetic delivery of IFN-α into CNS tumor sites [1].

While clinical development of adoptive transfer therapy using Type-1 CTLs specific for glioma-associated antigens (GAAs) and genetic delivery of IFN-α are feasible, efficient vaccine-based approaches may be developed as more logistically attractive alternatives, potentially by administration of a "natural" inducers of IFN, such as a Toll-like receptor (TLR)3 ligand, polyinosinic-polycytidylic acid (poly-IC) [2,3], stabilized with poly-lysine and carboxymethylcellulose (poly-ICLC) [4] as adjuvants.

The TLRs play essential roles in the initiation of innate and adaptive immunity. In mammals, the TLR family is composed of at least 11–12 members; and each TLR acts as a primary sensor of conserved microbial components, driving the induction of specific biological responses [5,6]. Among them, TLR3 is involved in the recognition of viral components, such as viral double-stranded (ds)RNA, and induces high levels of IFN-α/β [7-9]. Poly-IC serves as a TLR3 ligand and promotes the generation of Type-1 polarizing dendritic cells (DC) [10] and the induction of Type-1 Ag-specific immunity *in vivo *[11,12].

In the CNS and CNS tumors, recent studies have reported that both microglia [13] and astrocytes express TLR3 [3,14]. Especially for human astrocytes, TLR3 appears to be one of the most abundantly expressed TLRs [3], with TLR3 ligation inducing the production of pro-inflammatory cytokines such as IFN-α/β from astrocytes and microglia [3,14,15]. These data suggest that TLR3-mediated signaling may be key to expanding and directing systemic immunity into the CNS [16]. Indeed, poly-ICLC has been extensively evaluated as a single therapeutic agent in patients with malignant glioma, demonstrating clinical safety and feasibility [4]. Subsequent larger scale trials are currently underway and the confirmation of safety for poly-ICLC in these single-agent trials should support the continued use of this agent in prospective combinational vaccine trials.

As for integrin receptors involved in the adhesion of T-cells to endothelia, very late antigen-4 (VLA-4, the heterodimer of α4 and β1 integrins) has been demonstrated to confer T-cell homing to CNS inflammatory sites [17,18]. Calzascia *et al*. have recently demonstrated that up-regulation of VLA-4 on Ag-specific CTLs dictates efficient CNS-tumor tropism [19]. Given that IFN-α up-regulates VLA-4 on human T-cells [20], we hypothesized that poly-ICLC might enhance VLA-4 expression on vaccine-induced Ag-specific CTLs, thereby facilitating the CNS-tumor homing of these CTLs. We now show that poly-ICLC administration enhances the therapeutic efficacy of the Ag-specific vaccines, in part, by promoting the induction of Type-1 Ag-specific VLA-4+ CTLs in the i.c. M05 melanoma and GL261 glioma models.

## Methods

### Animals

C57BL/6 (H-2^b^) and ovalbumin (OVA)-specific T-cell receptor (TCR) transgenic OT-1 mice (C57BL/6-background) were purchased from Taconic (Germantown, NY). Pmel-1 mice (The Jackson laboratory, Bar Harbor, ME) are transgenic for a human(h)gp100_25–33 _– specific TCR, which cross-reacts with mouse(m) gp100_25–33_. Animals were handled in the Animal Facility at the University of Pittsburgh per an Institutional Animal Care and Use Committee-approved protocol.

### Cell lines

The mouse (H-2^b^) GL261 glioma cell line was kindly provided by Dr. Robert Prins (University of California, Los Angeles, CA), with the OVA-transfected B16 (M05) melanoma cell line kindly provided by Dr. Louis D. Falo, Jr. (University of Pittsburgh, PA). These cell lines were maintained in mouse complete medium [RPMI 1640 supplemented with 10 % heat-inactivated fetal bovine serum, 100 units/mL penicillin, 100 μg/mL streptomycin, and 10 mmol/L L-glutamine (all reagents from Life Technologies, Inc., Grand Island, NY)] in a humidified incubator in 5% CO_2 _at 37°C.

### Treatment of intracranial (i.c.) tumor-bearing mice with s.c. vaccination and i.m. poly-ICLC

Preparation of i.c. tumor-bearing mice was performed as previously described [1,21,22]. Briefly, 5 × 10^3 ^M05 or 5 × 10^4 ^GL261 cells in 2 μl PBS were stereotactically injected through an entry site at the bregma 2 mm to the right of the sagittal suture and 3 mm below the surface of the skull of anesthetized mice using a stereotactic frame. The animals received s.c. vaccinations with corresponding peptides emulsified in incomplete Freund Adjuvant (IFA) (Difco Laboratories, Detroit, Michigan, MI) and i.m. administrations with poly-ICLC (50 μg/injection; Oncovir Inc, Washington, DC) on indicated days. Animals were monitored daily after treatment for the manifestation of any pathologic signs. In some experiments, symptom-free survival was monitored as the primary endpoint, and in other experiments, treated mice were sacrificed on indicated days to evaluate immunological endpoints, such as brain infiltrating lymphocytes (BILs).

### Peptides and tetramers

All peptides, including H-2K^b^-restricted OVA_257–264 _(SIINFEKL), and mTRP2_180–188 _(SVYDFFVWL), H-2D^b^-restricted hgp100_25–33_(KVPRNQDWL) and mEphA2_671–679 _(FSHHNIIRL), I-A^b^-binding HBV core_128–140 _(TPPAYRPPNAPIL), were synthesized in the University of Pittsburgh Peptide Synthesis Facility, and were > 95% pure as indicated by analytical high-performance liquid chromatography and mass spectrometric analysis. Peptides were dissolved in phosphate-buffered saline (PBS)/10% dimethyl sulfoxide (DMSO) at a concentration of 2 mg/ml and stored at -20°C until use. Phycoerythrin (PE)-conjugated- H-2K^b^/TRP2_180–188 _tetramer was produced by the National Institute of Allergy and Infectious Disease (NIAID) tetramer facility at the Emory University Vaccine Center (Atlanta, GA). PE-H-2K^b^/OVA_257–264 _tetramer was purchased from Beckman Coulter Inc. (Fullerton, CA).

### Isolation of brain infiltrating lymphocytes (BILs)

BILs were isolated using the methods described previously [1,23]. In brief, mice were sacrificed by CO_2 _asphyxia, then perfused through the left cardiac ventricle with PBS. Brains were mechanically minced and cells from each brain were resuspended in 70% Percoll (Sigma, Saint Louis, MO), overlayed with 37% and 30% Percoll, then centrifuged for 20 min at 500 × g. Enriched BIL populations were recovered at the 70%-37% Percoll interface [19,24]. For *in vivo *inhibition of α_4_-integrin, mice received i.p. injections with anti-CD49d monoclonal antibodies (mAbs) (150 μg/mouse for R1-2 and 9C10; BD PharMingen, San Diego, CA) or 300 μg/mouse isotype rat IgG_2b_K, (clone A95-1; BD PharMingen, San Diego, CA) at the indicated time points.

### Antibodies and flow cytometry

TriColor (TC)-anti-CD3, fluorescein isothiocyanate (FITC)-anti-CD8α, PE-anti-CD8α, TC-anti-CD8, PE-anti-H-2K^b^, FITC-anti-CD49d (α4-integrin), FITC-anti-TCRvβ13, purified anti-CD49d (clones R1-2 and 9C10) mAbs were purchased from BD PharMingen (San Diego, CA). PE-anti-TLR3 was purchased from eBioscience. IFN-β-neutralizing mAb (MIB-5E9.1) and the corresponding, functional grade, purified Armenian hamster IgG (eBio299Arm) were obtained from BioLegend and eBioscience, respectively. Single-cell suspensions of splenocytes (SPCs), lymph node (LN) cells and BILs were stained with 10 μg/ml PE-labeled MHC-peptide-specific tetramers in PBS containing 1% BSA for 15 min at room temperature, then washed once and stained with TC-anti-CD3 or FITC-anti-CD8 mAbs. For intracellular IFN-γ staining, cells were surface-stained with PE-anti-CD8 mAb, washed, fixed, and then permeabilized with Cytofix/Cytoperm buffer (BD PharMingen) before staining with anti-IFN-γ mAb (BD PharMingen). Flow-cytometric analyses were performed using Coulter EPICS cytometer for lymphocyte-gated cell populations (Beckman Coulter, Fullerton, CA).

### *In vivo *CTL assay

C57BL/6 mice were immunized s.c. with the OVA (100 μg) and the helper HBV Core_128–140 _(150 μg) peptides emulsified in IFA and i.m. poly-ICLC (50 μg/ml) on days 0 and 7. On day 14, erythrocyte-depleted SPC and lymph node cells from naïve C57BL/6 mice were either: 1) pulsed with 10^-6 ^M OVA-peptide, incubated at 37°C for 1 hour, and labeled with a high concentration of Carboxyfluorescein diacetate, succinimidyl ester (CFDA SE; CFSE) (2.5 μM) (CFSE^high ^cells); or 2) pulsed with no peptide and labeled with a low concentration of CFSE (0.25 μM) (CFSE^low ^cells). Then, equal numbers of cells from each populationwere mixed and i.v. infused into immunized mice (1 × 10^7 ^cells in 200 μl PBS/mouse). At 4 hrs after i.v. infusion, SPC were evaluated for the ratio of CFSE^low ^to CFSE^high ^cells by flow cytometry. To calculate specific lysis, the following formula was used: ratio = (percentage CFSE^low^/percentage CFSE^high^). Percentage specific lysis = [1 – (ratio unprimed/ratio primed) × 100] [25].

### *In vitro *CTL assay

C57BL/6 mice were immunized s.c. with synthetic, GL261-derived CTL epitopes (50 μg TRP-2_180–188 _and hGP100_25–33_, 100 μg mEphA2_671–679_) and the helper peptide (150 μg HBV Core_128–140_) emulsified in IFA (GAA-vaccine) in combination with i.m. poly-ICLC on days 0 and 7. SPCs were harvested on day 14, and *in vitro *re-stimulated for 5 days with IL-2 (20 IU/ml) and each of (5 μg/ml) three GAA peptides before SPCs were tested for their lytic activity against GL261 glioma cells and control, GAA-negative EL-4 cells in a 4-hr standard ^51^Cr-release assay as previously described [22].

### Cytokine release assay

Single cell-suspended BILs, SPCs and LN cells were restimulated with OVA_257–264 _peptide (5 μg/ml) in the presence of rhIL-2 (20 U/ml) for indicated time. Culture supernatants were assessed for mIFN-γ using a specific ELISA kit (BD PharMingen, San Diego). IFN-inducible protein (IP)-10 production levels from tumor cells were determined by mIP-10 ELISA kit (R&D, Minneapolis, MN).

### Histological analyses of i.c. tumors

Perfusion-fixed brains were obtained from mice treated with GAA-vaccine and poly-ICLC on day 90 after the tumor inoculation. The brains were then embedded in optimal cutting temperature compound (4583; Sakura Finetek), frozen at -80°C, and thin coronal sections [20 μm for Luxol Fast Blue (LFB) and 5 μm for Hematoxylin & Eosin (H&E)] were made using a cryostat. Sections were stained with H&E to evaluate the overall infiltration of mononuclear immune cells, or with LFB to evaluate the demyelination.

### Statistical analysis

Survival data were compared using Log rank test. Comparative T-cell responses were analyzed by one way analysis of variance for comparing means of three or more variables, ANOVA).

## Results

### EphA2 is an antigen associated with GL261 glioma, but not normal brain

**Figure 1:**
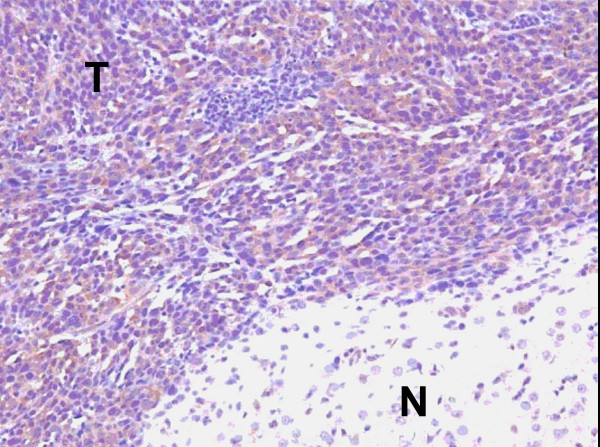
**High-level expression of EphA2 is restricted to GL261 glioma in the mouse brain**. Paraffin embedded tissue sections prepared from the brains of C57BL/6 mice bearing day 14 GL261 glioma in the right frontal lobe were stained with anti-EphA2 monoclonal antibody (C-20 Ab; Santa Cruz Biotechnology, Inc). After washing, sections were incubated with biotinylated goat anti-rabbit IgG (Vector Laboratories), followed by avidin-biotin-complex peroxidase (Vectastain ABC kits; Vector Laboratories). Reaction products were developed using a Nova Red substrate kit (Vector Laboratories) giving rise to red-brown deposits. The sections were also counter-stained with hematoxylin (blue). The letter "T" in the figure indicates tumor tissue, with the letter "N" in the figure indicating normal brain tissue. Original magnification; × 20.

GL261 glioma cells (H-2^b^) express well-characterized CTL epitopes, such as the H-2D^b^-restricted mgp100_25–33 _and the H-2K^b^-restricted TRP-2_180–188 _peptides [26]. In addition, we recently identified a novel T cell epitope H-2D^b^-restricted mEphA2_671–679_, derived from the receptor tyrosine kinase mEphA2, which is overexpressed in human [27] and murine gliomas, including GL261 [28]. We hereby define these three antigens (mgp100, TRP-2 and EphA2) as the glioma-associated antigens (GAAs) in the GL261 glioma cells. As demonstrated in Fig. 1, immuno-staining revealed a high-level expression of EphA2 in the GL261 tumor; whereas the normal brain did not demonstrate significant staining. These results confirm the Western Blot analyses reported in our previously published study [28]. In the case of the M05 melanoma model system, the OVA_257–264 _peptide represents an additional "tumor" epitope [1].

### I.m. poly-ICLC administration enhances specific CTL responses against OVA and GAA-derived CTL epitopes

**Figure 2:**
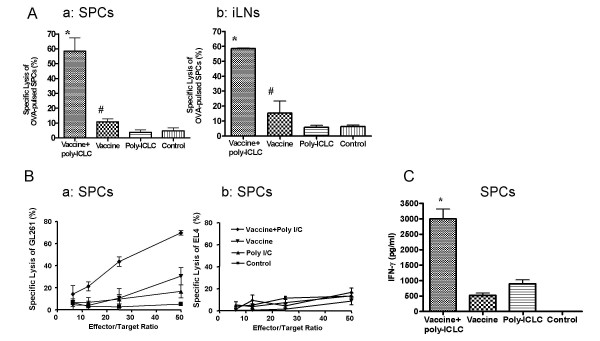
**Poly-ICLC administration enhances vaccine-induced specific CTL generation in vivo**. (A), C57BL/6 mice received OVA-vaccines with or without i.m. poly-ICLC on days 0 and 7 (n = 3/group). OVA-specific CD8+ T cell activities in SPC (a) and draining inguinal LN cells (b) were evaluated by *in vivo *CTL assays on day 14. *P < 0.01 for the combination group compared with poly-ICLC alone or control group; and P < 0.05 for the combination group compared with vaccine alone group. #P > 0.05 for the group receiving vaccine alone compared with poly-ICLC alone or control group. (B), C57BL/6 mice received GAA-vaccines with or without i.m. poly-ICLC on days 0 and 7. SPCs were harvested on day 14. Following a 5-day *in vitro *stimulation with 20 IU/ml IL-2 and three GAA peptides, SPCs were tested for their lytic activity against GL261 glioma cells (a) or EL4 cells (b) in a 4-hr standard ^51^Cr-release assay. (C), C57BL/6 mice received EphA2-vaccines with or without i.m. poly-ICLC on days 0 and 7. On day 14, SPC were harvested, *in vitro *stimulated with low-dose (20 IU/ml) hIL-2 and mEphA2_671–679 _for 5 days prior to performance of mouse IFN-γ specific ELISA using culture-supernatants. *P < 0.001 compared to vaccine alone, poly-ICLC alone and the control groups. For (A-C), n = 3/group, and data represent results from one of 3 independent experiments performed with similar results obtained. Error bars represent standard deviation (SD).

We sought to determine whether poly-ICLC administration could enhance the induction of specific CTL response to peptide epitopes employed in vaccines. *In vivo *CTL assays against OVA_257–264_-peptide demonstrated that concurrent i.m. poly-ICLC and OVA peptide-based vaccine administration yielded remarkably enhanced induction of specific CTL responses in both SPCs and draining LN cell populations (Fig. 2A). Furthermore, i.m. poly-ICLC administration augmented anti-GL261 CTL responses induced by vaccinations with 3 endogenous GAAs, mEphA2_671–679_, hgp100_25–33_and TRP-2_180–188 _in addition to the HBVcore_128–140 _T-helper epitope (GAA-vaccines) (Fig. 2Ba). EL-4 thymoma cells that do not express these GAAs were used as negative control target cells, and were poorly recognized (< 10% lysis) in all groups (Fig. 2Bb), supporting the specificity of the vaccine-induced CTL responses. Poly-ICLC administration also enhanced the SPC-production of IFN-γ following vaccinations with mEphA2_671–679 _(Fig. 2C).

### Poly-ICLC facilitates the infiltration of Ag-specific T cells into the CNS tumor site

**Figure 3:**
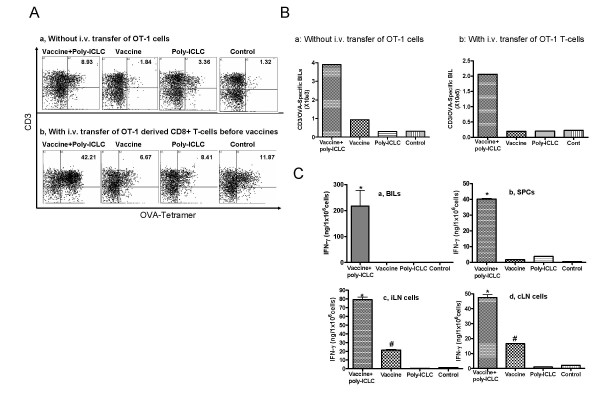
**Poly-ICLC administration promotes the infiltration of Type-1 Ag-specific T cells into CNS rumor sites**. (A-C), mice bearing day 10 M05 tumors were immunized with either: OVA_257–264_vaccines plus poly-ICLC, OVA_257–264 _vaccines only, mock vaccines plus poly-ICLC, or control mock vaccines on days 10 and 15. In the experiments depicted in (A-b, B-b, C), mice also received i.v. injections of 5 × 10^6 ^OT-I mice-derived naïve SPCs and LN cells on day 10 prior to the first immunization. On day 16, the mice were sacrificed, and BILs from tumor-bearing hemisphere were analyzed for the presence of OVA_257–264_-specific T-cells using TC-labeled anti-mouse CD3 mAb and PE-labeled OVA_257–264_-specific tetramer. N = 5 mice/group. BILs from the same group were pooled and compared between groups. (A), numbers represent the percentage of CD3^+^/OVA tetramer^+ ^cells in lymphocyte-gated BILs. (B), total numbers of CD3^+^/OVA tetramer^+ ^BILs per mouse with (b) or without (a) adoptive transfer of naïve OT-1 mouse-derived SPCs and LN cells. (C), expression of IFN-γ by isolated BILs (a), SPCs (b), ipsilateral inguinal (c), and cervical (d) LN cells. Aliquots of 1.0 × 10^6^/ml isolated lymphocytes were cultured with 5 μg/ml OVA_257–264 _and 20 IU/ml rhIL-2 for 6 h (BILs) or 5 days (for SPCs and LN cells), and IFN-γ in the supernatant was measured by specific ELISA. For BILs (a), *P < 0.05 compared to all other groups. For SPCs (b), *P < 0.001 compared to all other groups. For iLNs (c) and cLNs (d), *P < 0.001 for the combination group compared to all other groups, #P < 0.01, for the vaccine alone group compared to the control or the poly-ICLC alone group. *Columns*, mean of three wells in 96 well plate; *Error bars*, SD. Representative of 2 and 4 independent experiments with similar results, for (a) and (b), respectively.

To determine whether poly-ICLC administration enhanced the infiltration of brain tumors by vaccine-induced OVA_257–264_- specific CTLs, C57BL/6 mice bearing intracranial (i.c.) M05 tumor received vaccinations using OVA peptide with or without poly-ICLC for two cycles prior to isolation of BILs. The percentage (Fig. 3A) and the absolute numbers of CD3^+^/OVA tetramer^+ ^BILs per mouse (Fig. 3B) were then analyzed by flow cytometry. As displayed in Figs. 3Aa and 3Ba, there were relatively few OVA-tetramer/CD3-double positive BILs in mice that had received mock-vaccination or poly-ICLC alone. Even though the percentage of OVA-tetramer/CD3-double positive cells appeared to be slightly elevated as a result of poly-ICLC treatment alone (Fig. 3Aa), the actual number of these cells was still low, due to the low total number of BIL obtained from this group (Fig. 3Ba). Although OVA-vaccines appeared to have increased the total number of OVA-specific T-cells moderately, due to the increase of total CD3^+ ^lymphocytes in this group compared to groups treated with poly-ICLC alone or mock-treated mice (Fig. 3Ba), the numbers of OVA-tetramer/CD3-double positive cells remained less than 1000/mouse in these three groups in 2 independent experiments. In contrast, the addition of poly-ICLC to the OVA-vaccine regimen remarkably increased tumor-infiltration by OVA-specific responder T cells (3,900/mouse).

To improve the sensitivity and reliability of flow-cytometry based enumeration of BILs, in parallel experiments, i.c. M05 bearing mice received i.v. transfer of naïve OT-1 mice-derived SPCs and LN cells prior to the first immunization. As demonstrated in Figs. 3Ab and 3Bb, the combination therapy with OVA-vaccines and i.m. poly-ICLC administrations resulted in a remarkable increase in the percentage (Fig. 3Ab), and in the absolute numbers (2.06 × 10^5^/mouse) (Fig. 3Bb) of OVA-specific CD3^+ ^BILs per mouse compared to other groups including mice receiving OVA-vaccines alone (2.05 × 10^4^/mouse).

To evaluate whether the increased numbers of OVA-reactive T-cells were also evident systemically, we also harvested lymphocytes from the spleen, draining inguinal LNs and cervical LNs, and evaluated these populations for their frequencies of OVA-reactive CD8^+ ^T cells. The percentage of OVA_257–264_-reactive CD3^+ ^T cells among total lymphocyte gated populations ranged from 0.54 – 1.05% and 0.34–0.65% in mice treated with both OVA-vaccines plus poly-ICLC, vs. mice treated with OVA-vaccines alone, respectively (data not shown). Hence, poly-ICLC administration in combination with OVA-vaccination preferentially promoted the infiltration of vaccine-induced OVA-specific T-cells into brain tumor sites.

To assess the functional status of BILs, freshly-isolated BILs were evaluated for expression of IFN-γ following a brief (6 hrs) *in vitro *re-stimulation with the OVA_257–264_-peptide and low-dose rhIL-2 (Fig. 3Ca). BILs obtained from mice receiving both OVA-vaccines and poly-ICLC produced high levels IFN-γ, whereas IFN-γ levels were undetectable in all other groups. SPCs and LN cells obtained from mice receiving the combination therapy produced higher levels of IFN-γ than those obtained from other groups following 96 hr *in vitro *stimulation (Fig. 3Cb–d) (P < 0.001). These results suggest that poly-ICLC delivery enhances Type-1 (i.e. IFN-γ expressing) function of vaccine-stimulated T cells, especially within the i.c. tumor-microenvironment.

### Poly-ICLC treatment up-regulates VLA-4 expression on OVA-specific T cells that dictates their CNS-tumor homing capacity

**Figure 4:**
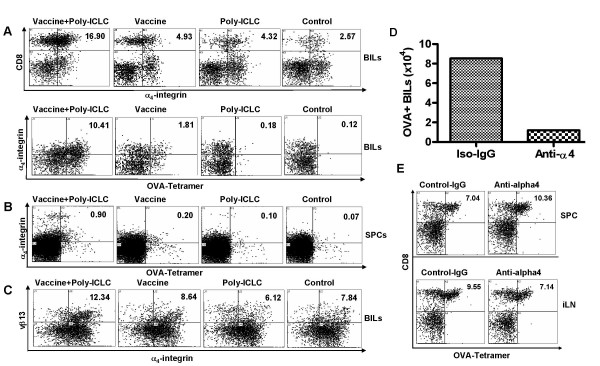
**Poly-ICLC enhances the T-cell expression of α4-integrin (CD49d), which confers efficient CNS-tumor homing of Ag-specific T-cells**. (A and B), C57BL/6 mice bearing day 10 i.c. M05 tumors received 5 × 10 ^6 ^naive OT-1 mouse-derived T-cells, then OVA-vaccines and/or poly-ICLC administrations on days 10 and 15. BILs (A) and SPCs (B) were harvested on day 16, and evaluated for the α_4_-integrin expression on CD8^+^, OVA-tetramer^+ ^cells by flow cytometry. (A), numbers represent the percentage of α_4_-integrin^+^/CD8^+ ^(upper panel), α_4_-integrin^+^/OVA^+ ^cells (lower panel) in lymphocyte-gated BIL populations. (B), numbers represent the percentage of α_4_-integrin^+^/OVA^+ ^cells in lymphocyte-gated SPC populations. (C), C57BL/6 mice bearing day 15 i.c. GL261 tumors received 5 × 10 ^6 ^naive Pmel-1 mouse-derived T cells, then hgp100-vaccines and/or poly-ICLC administrations on days 15 and 20. BILs were harvested on day 21, and evaluated for the α_4_-integrin expression on CD8^+^/TCRvβ13^+ ^cells by flow cytometry. Numbers represent the percentage of α_4_-integrin^+^/TCRvβ13^+ ^T-cells in lymphocyte-gated populations. (D and E), mAb-mediated blockade of α4-integrin inhibited the CNS-tumor infiltration of OVA-specific T-cells, while not depleting Ag-reactive T-cells systemically. C57BL/6 mice bearing day 10 i.c. M05 tumors received i.p. injections of anti-α 4-integrin mAbs (R1-2, 150 μg/mouse and 9C10, 150 μg/mouse), or control isotype mAb (rat IgG_2b_K, clone, A95-1, 300 μg/mouse) at 2 hrs before i.v. adoptive transfer of 5 × 10^6 ^naïve OT-1 mouse-derived T-cells and subsequent OVA-vaccination and poly-ICLC administration. On day 13, the mice received the 2^nd ^OVA-vaccination and poly-ICLC administration at 2 hrs following the 2^nd ^i.p. mAb injections. BILs, SPC and lymphocytes from draining inguinal (i)LNs were harvested on day 16. Numbers of CD3^+^/OVA tetramer^+ ^BILs per mouse (D), and the presence of CD8^+^, OVA-tetramer reactive T cells in iLN and SPC are depicted (E). Data are representative of 3 independent experiments with similar results.

Efficient CNS-tumor homing of Ag-specific T-cells activated by Ag-specific vaccines and poly-ICLC administration led us to determine whether poly-ICLC administration induces qualitative changes in the phenotype of vaccine-activated OVA-reactive T cells particularly in the context of homing receptors involved in brain tumor-tropism. Based on a recent study by Calzascia *et al*. demonstrating that up-regulation of VLA-4 expression on Ag-specific CTLs confers efficient CNS-tumor tropism [19], we evaluated expression of CD49d (Integrin α4 chain), a subunit for the VLA-4, on BILs (Fig. 4A) and SPCs (Fig. 4B) obtained from mice receiving OVA-vaccines and/or i.m. poly-ICLC administrations. As depicted in Fig. 4A, *in vivo *administration of poly-ICLC remarkably increased the number of α_4_-integrin (CD49d)^+ ^OVA-tetramer binding CD8^+ ^cells in BILs. Although the percentage was lower compared to BILs, the combination regimen also increased the numbers of α_4_-integrin (CD49d)^+ ^OVA-tetramer binding cells in SPCs (Fig. 4B). These cells also expressed β_1_-integrin (CD29), but did not express detectable α_4_β_7 _integrin heterodimers (data not shown), indicating that α_4_-integrin (CD49d) is solely expressed in VLA-4 complexes.

We next performed parallel experiments using i.c. GL261-bearing mice treated by adoptively transfer of naïve Pmel-1 derived CD8^+ ^cells followed by vaccination with gp100 peptides and poly-ICLC administration. We observed results similar to the M05 model, with increased i.c. glioma-infiltration by α_4_-integrin/TCR-Vβ13 double-positive cells as a result of this treatment regimen (Fig. 4C).

VLA-4 allows activated T-cells to adhere to vascular cell-adhesion molecule (VCAM)-1^+ ^endothelial cells [17,18]. Therefore, these data suggest the possibility that VLA-4 may play a significant role in the CNS-tumor homing of CTLs induced by vaccine plus poly-ICLC co-treatment. To address this hypothesis, we evaluated the effects of specific mAb-mediated blockade of the VLA-4/VCAM-1 interaction (Fig. 4D). Before these *in vivo *experiments, we confirmed that each of two anti-α_4_-integrin mAbs (Clones R1-2 and 9C10), and especially the combination of these two mAbs, effectively blocked the binding of activated T-cells to plastic plates coated with VCAM-1 (Sasaki *et al*. submitted). C57BL/6 mice bearing day 10 i.c. M05 tumors received i.p. injections of anti-α_4_-integrin mAbs (R1-2, and 9C10), or control isotype mAb (rat IgG_2b_K, clone, A95-1) 2 hrs prior to i.v. adoptive transfer of 5 × 10^6 ^naïve OT-1 mouse-derived T-cells, which was immediately followed by OVA-vaccination and poly-ICLC administration. On day 13, i.p. mAb injections and vaccinations were repeated. BILs were harvested on day 16, and evaluated by flow cytometry. As depicted in Fig. 4D, the functional blockade of α_4_-integrin by specific mAbs diminished the CNS-tumor homing of OVA-specific T-cells. Treatment with mAbs did not affect the peripheral expansion of OVA-reactive T-cells, as the numbers of OVA-tetramer reactive T-cells in lymphoid organs were not significantly altered by mAb treatments (Fig. 4E). These data suggest that the mAb-mediated blockade of α_4_-integrin inhibits the tumor-homing of these effector cells, but not their induction or expansion. Even though efficient tumor-homing by Ag-specific CD8^+ ^T cells most likely results from the orchestrated cooperation of multiple receptor-ligand combinations [29], these results indicate that poly-ICLC induced VLA-4 expression on vaccine-induced CTL dominantly influences their capacity to infiltrate into CNS tumors.

### Combination vaccine + poly-ICLC therapy is an effective treatment for mice bearing CNS glioma and induces long term anti-tumor protection

**Figure 5:**
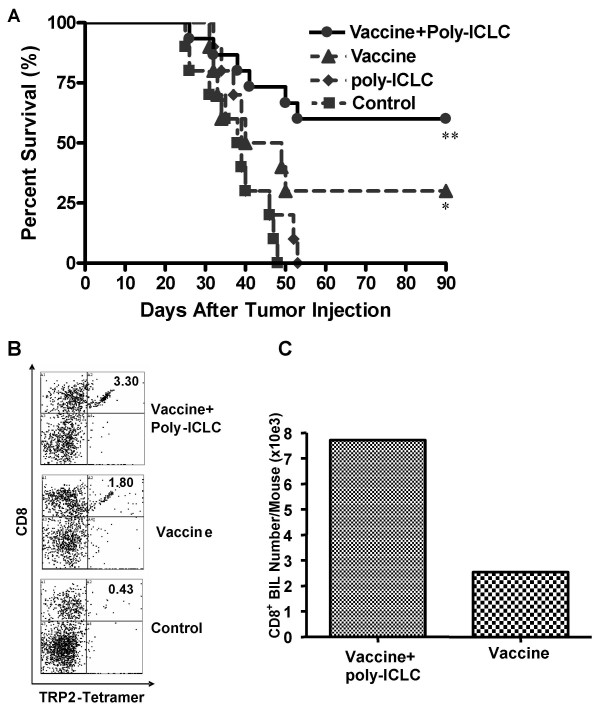
**GAA-vaccines in combination with i.m. poly-ICLC administrations induce long-term anti-GL261 protective immunity**. (A), C57BL/6 mice received s.c. GAA-vaccines with or without i.m. poly-ICLC on days -14 and -7. On day 0, mice received i.c. inoculations of 1 × 10^5 ^GL261 cells, with survival subsequently monitored. **P = 0.003 for the mice receiving combination treatments compared with the control group. *P = 0.0521 for the mice receiving GAA-vaccines alone compared with the control group (Log rank test). (B and C), mice that survived for 90 days following GAA-vaccines and i.m. poly-ICLC or GAA-vaccine alone in (A) were re-challenged with 5 × 10^4 ^GL261 in the contralateral hemisphere of the brain (n = 3/group). As controls, naïve mice received the same number of GL261 cells. BILs were harvested from the tumor-bearing hemisphere at 7 days after tumor re-challenge, and stained with TC-anti-CD8 and PE-H-2K^b^/TRP2_180–188_-specific tetramer. (B), numbers represent the percentage of CD8^+^/TRP-2 tetramer^+ ^cells in lymphocyte-gated BILs in each group. (C), numbers of viable CD8^+ ^BILs per mouse.

To evaluate whether the enhanced, systemic and local Ag-specific T-cell responses stimulated by the combination therapy would translate into a more clinically-relevant model, C57BL/6 mice were pre-immunized with GAA-vaccines on days -14 and -7, with or without poly-ICLC administration, before they received i.c. injection of 5 × 10^4 ^GL261 cells in the right hemisphere on day 0. As depicted in Fig. 5A, all mice receiving mock treatments died by day 47. Treatment with poly-ICLC alone had no therapeutic effect when compared to the mock vaccine group (P > 0.05). Although GAA-vaccination alone resulted in long-term (> 90 days) survival in 3 of 10 mice, this level of protection did not reach statistical significance when compared to the control, mock treatment group (p = 0.0521). In contrast, addition of poly-ICLC to the GAA-vaccine protocol improved survival, with 9 of 15 animals still alive on day 90, a statistically significant benefit when compared with the control (P = 0.003) group.

To further determine whether i.c. tumor-bearing mice immunized with GAA-vaccines and poly-ICLC exhibit long-term anti-tumor memory immune response, survivors in experiments in Fig. 5A were re-challenged with 5 × 10^4 ^GL261 cells in the contralateral hemisphere of the brain on day 90 after the initial tumor inoculation. As a control group, non-immunized age-matched mice also received the same i.c. GL261 cell-inoculations. On day 7 after tumor re-challenge, mice were sacrificed, and BILs were harvested. As shown in Fig. 5B, flow-cytometric analyses of BILs revealed an elevated percentage of H-2K^b^/TRP-2_180–188 _tetramer reactive CD8^+ ^T cells in the vaccinated, long-term survivors when compared to BILs obtained from control non-immunized GL261-bearing mice. Furthermore, mice received the combinational regimen exhibited approximately 3-fold higher numbers of CD8^+ ^BILs when compared to mice treated with GAA-vaccines alone (Fig. 5C). These results strongly suggest that the GAA-vaccines in combination with poly-ICLC, can induce effective, long-term memory responses that are protective against i.c.GL261 progression.

### Luxol Fast Blue (LFB) and Hematoxylin and Eosin (H&E) staining suggest the lack of autoimmune encephalitis in mice treated with GAA-vaccines and poly-ICLC

**Figure 6:**
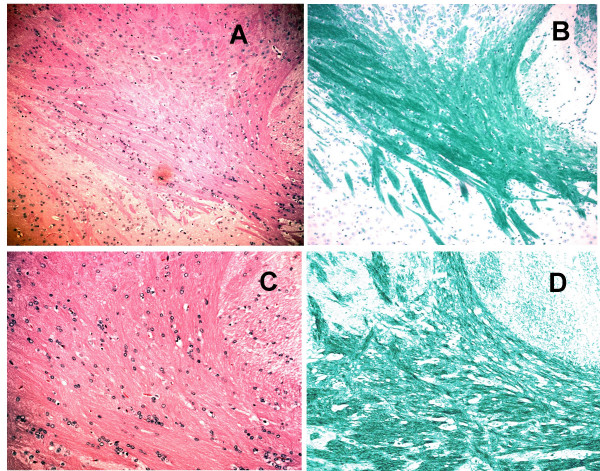
**H&E and LFB staining of brain sections reveal the absence of pathologic autoimmunity**. Perfusion-fixed brains were obtained from GL261-bearing mice treated with GAA-vaccine and poly-ICLC on day 90 after the tumor-inoculation. Frozen sections were stained with LFB (B and D). Cryostat sections were also stained with H&E to evaluate the overall infiltration of mononuclear immune cells (A and C). Images were taken from the basal ganglia. The thick bundle strongly stained with LFB indicates internal capsule. All images were obtained from the corresponding visual fields. The original magnifications are × 10 (for A and B), and × 20 (for C and D). There was no evidence of demyelination, hemorrhage, or pathological immune cell infiltration throughout the brain.

To determine whether the combination therapy with GAA-vaccines and poly-ICLC administration induces demyelination and/or autoimmune encephalitis, we evaluated brain sections obtained from treated mice by LFB staining and H&E staining using standard protocols [30]. Fig. 6 depicts representative stained sections derived from mice treated with the combinational regimen. Highly-myelinated structures, such as corpus callosum and internal capsule, were densely stained with LFB. There was no evidence of demyelination or abnormal immune cell infiltration throughout the brain. We also examined control mice treated with GAA-vaccines alone or with mock-vaccines, and found no evidence of autoimmunity in these animals (data not shown).

## Discussion

Our current study is novel and significant for several reasons. We report that: 1) poly-ICLC can serve as a safe and effective adjuvant to improve the efficacy of Ag-specific peripheral vaccinations in mouse CNS tumor models, including the GL261 glioma; 2) VLA-4 expression on CTLs plays a significant role in the efficient CNS tumor infiltration of Ag-specific CTLs induced by the vaccinations and poly-ICLC treatment; 3) efficient CNS tumor homing of Ag-specific T-cells was also associated with Ag-specific IFN-γ production of BILs, suggesting the improved Type-1 function of tumor infiltrating CD8+ T effector cells as a result of poly-ICLC co-administration. Our data with both GL261 glioma and M05 melanoma models provide us with insights that may be applicable to immunotherapy for both primary and metastatic CNS tumors.

**Figure 7:**
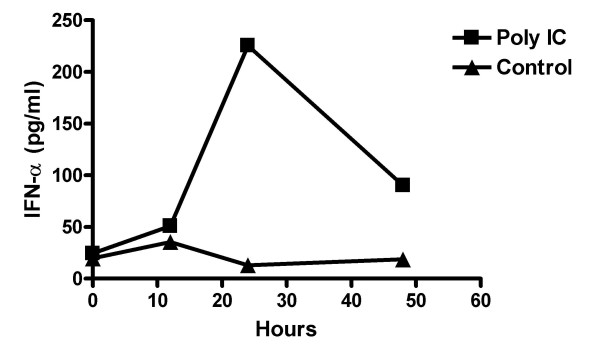
**Poly-ICLC administration induces IFN-α in serum of treated mice.** Peripheral blood samples were drawn from the tail vein at 0, 12, 24 and 48 hrs following i.m. poly-ICLC (50 μg) treatment. Serum IFN-α levels were determined using specific ELISA (Endogen, Rockford, IL). The average values of 3 mice in treated (■) or non-treated (▲) mice are depicted (n = 3/group). Systemic induction of IFN-α in serum peaked at 24 hrs following the poly-ICLC injection, whereas control mice with no poly-ICLC administration did not display any elevation in IFN-α levels.

With regard to the underlying mechanisms supporting the enhanced induction of Ag-specific CTLs by TLR3 stimulation in our systems, recent studies using poly-IC in murine tumor models have demonstrated that poly-IC increases the transcription of the anti-apoptotic molecules Bcl-3 and Bcl-xL in T cells, thereby inhibiting their apoptosis [12], and that poly-IC-induced activation of natural killer (NK) cells, which is dependent on host-derived IFN-γ, IL-12 and IL-15, is at least partially responsible for the adjuvant effects of poly-IC [11]. In this referenced study, it was also noted that the timing of poly-IC administration was critical. Enhanced Ag-specific CD8^+ ^T cell responses were observed only when poly-IC was administered within 4 hrs following peptide vaccination. Although these studies provide valuable insights regarding the mechanisms underlying the adjuvant effects of poly-ICLC, we have observed prolonged elevation of serum IFN-α response (at least for 48 hrs) (Fig. 7) than reported data using poly-IC [11], most likely due to the improved chemical stability of poly-ICLC when compared to poly-IC. Induction of systemic IFN-α is likely to play a critical role in our observed data because of its ability to mature DCs [10,31,32], promote cross-priming [33] and sustain the survival of activated T-cells [34,35]. Indeed, TLR3-mediated stimulation has been reported to enhance CD8^+ ^T-cell responses against OVA-protein in an IFN-α/β signaling-dependent manner [36]. We are currently evaluating the role of the host IFN-α/β responsiveness in the promotion of GAA-specific CTLs using IFN-α/β receptor^-/- ^mice as recipients in our i.c. tumor models.

Our data demonstrating that VLA-4 expression on vaccine-induced CTLs plays a major role in their efficient CNS-tumor homing confirm a recent study using an alternate CNS tumor model [19]. Our data also suggest that poly-ICLC administration promotes not only the systemic induction of Ag-specific CTLs, but alters their profile of surface receptors expressed allowing for the efficient CNS-tumor homing. The precise mechanism(s) as to how poly-ICLC promotes VLA-4 expression on T-cells requires further investigation. Both Type-1 helper T-cells [i.e. Th1; [37]] and Type-1 CD8+ T cells [i.e. Tc1; [38]] express higher levels of VLA-4 when compared to their Type-2 counterparts. With regard to direct cytokine signals that promote VLA-4 expression, IFN-α appears to up-regulate VLA-4 on human T cells [20]. Therefore, it is likely that poly-ICLC induces VLA-4 expression on T-cells by supporting a Type-1 cytokine milieu that includes IFN-α. Experiments to evaluate this hypothesis using IFN-α/β receptor^-/- ^mice appear warranted.

Our studies evaluating IFN-γ production from freshly-isolated BIL and *in vitro *stimulated LN cells suggested that poly-ICLC administration systemically promotes Ag-specific, Type-1 T cell responses. This is likely to be mediated through the activation of NK and antigen-presenting cells (APCs) by poly-ICLC, since these cells express TLR3 [39-41], and these cells co-stimulate each other in the presence of poly-IC in promoting both the Type-1 polarizing functions of DC [42] and the enhanced cytotoxic ability of NK cells [43]. As IFN-γ was produced in response to MHC class I-restricted, OVA peptide stimulation [44,45], these results strongly suggest that poly-ICLC promotes specific Type-1 CTL (Tc1) responses that favor efficient tropism into CNS tumors, as well as therapeutic efficacy [1].

Our results showing high level IFN-γ production from BILs derived from mice treated with vaccination + poly-ICLC administration suggest the possibility that i.m. delivery of poly-ICLC directly stimulates the functions of APCs and other immune cells infiltrating the CNS tumors, such as microglia, thereby providing the secondary stimulation of vaccine-induced Ag-specific CTLs within the CNS-tumor environment. Experiments evaluating this hypothesis are currently underway.

**Figure 8:**
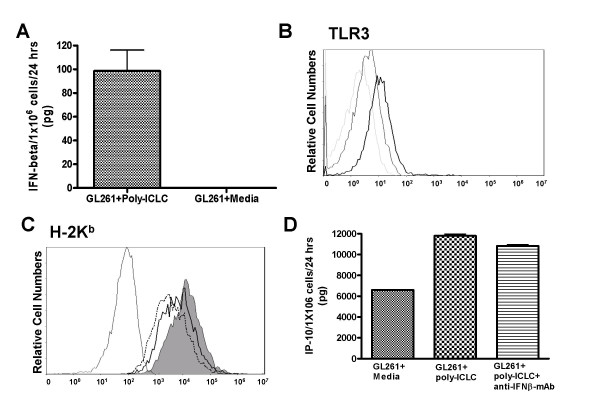
**Poly-ICLC stimulates GL261 glioma-expression of IFN-beta, TLR3, MHC class I and IP-10**. *In vitro *cultured GL261 cells were treated with or without 50 μg/ml poly-ICLC for 24 hrs, and evaluated for expression of IFN-β in culture-supernatant by specific ELISA (A), surface TLR3 (B) and H-2K^b ^(C) by flow-cytometry, and for production of IP-10 in culture-supernatant by specific ELISA (D). (B), *Dashed thin line: *isotype control antibody, *Thin line: *TLR3 expression without poly-ICLC treatment, *Bold line: *TLR3 expression in the presence of poly-ICLC. (C), *Black thin line: *isotype control antibody, *Dashed thin line*: H-2K^b ^expression without poly-ICLC treatment, *Filled grey area: *H-2K^b ^expression with poly-ICLC treatment and control isotype-IgG, *Black thick line: *H-2K^b ^expression with poly-ICLC treatment and neutralizing anti IFN-β mAb.

*In vitro *stimulation of cultured GL261 cells with poly-ICLC induced secretion of IFN-β and IP-10, as well as upregulation of the TLR3 and MHC class I (H-2K^b^) molecules (Fig. 8). Interestingly, the upregulation of H-2K^b^, but not IP-10, was at least partially dependent upon poly-ICLC-induced IFN-β signaling as demonstrated by blockade of Type-I IFN (IFN-β) signaling using IFN-β specific mAb (Fig. 8). As upregulated MHC class I expression directly mediates recognition by Ag-specific T-cells in inflammatory conditions [46,47], these results suggest that poly-ICLC treatment may directly sensitize GL261 glioma cells to the CTL-mediated immuno-surveillance via induction of Type-I IFN and the resultant upregulation of MHC class I and IP-10 production. In our previous study [1], IFN-induced chemokine IP-10 played a critical role in the efficient recruitment of Tc1 cells capable of mediating therapeutic efficacy; therefore, it is likely that the efficacy of poly-ICLC assisted vaccines observed in our current study also relies on IP-10. In addition to human astrocytes [3], our preliminary data indicate U87 human glioma cells also express functional TLR3 (unpublished observations), suggesting the clinical relevance of direct TLR3 stimulation on glioma cells. We are currently evaluating whether i.m. administered poly-ICLC can directly stimulate GL261 tumors injected into the brain.

In contrast to GL261, M05 cells did not express detectable levels of TLR3, nor could they directly respond to poly-ICLC *in vitro *(data not shown), although they did express high levels of IP-10 in response to IFN-γ treatment [1]. Based on the IFN-γ expression by BILs in i.c. M05 tumors following poly-ICLC assisted vaccinations, it is possible that BIL-derived IFN-γ production may have induced M05-tumor expression of MHC class I molecules and secretion of IP-10. Collectively, our data suggest that poly-ICLC may promote the attraction of vaccine-induced CTLs via direct and/or indirect induction of chemokines, such as IP-10, and promote the target tumor-cell recognition by the CTLs via upregulated MHC class I regardless of the endogenous TLR3 status of the tumor cells.

With regard to a preferred vaccine formulation for the treatment of gliomas, we believe that use of multiple peptides has a clear advantage over strategies with a single peptide. Given the marked antigenic heterogeneity of gliomas, immunotherapy with a single tumor-specific T-cell epitope might merely promote transient stabilization of disease, prior to the progression of antigen-loss variants [48]. We therefore remain dedicated to broaden the list of available human CTL-epitopes for integration into multi-epitope-based vaccine strategies for glioma therapy [23,27]. As the three GAAs we employed in the current study represent self-Ags with no tumor-specific mutations, they appear reflective of most defined human cancer Ags/epitopes (reviewed in [49]). Therefore, we believe that our GL261 tumor model that integrates therapies based on these GAA-epitopes will continue to serve as a highly clinically relevant model that limits concerns for inherited immunogenicity and genetic drifts associated with chemically-induced murine glioma models [50].

Our studies also indicate the lack of demyelination or increased immune-cell infiltrate in the normal CNS using LFB and H/E staining, which supports the absence of severe CNS-autoimmunity as a collateral pathologic event that might be associated with our combinational treatment regimen. Given the caveat that these data reflect those of short-term treatment, however, longer-term assessments are clearly warranted and we recognize that IHC will be invaluable in identifying the nature of infiltrating immune cells.

## Conclusion

Taken together, our current study demonstrated that poly-ICLC assisted Ag-specific vaccines may represent an efficient and safe therapeutic approach for CNS tumors. Future studies will attempt to elucidate detailed mechanisms underlying the enhanced induction of Type-1 Ag-specific T-cell responses by this treatment strategy.

## Abbreviations

BIL, brain infiltrating lymphocyte; CLN, cervical lymph node; i.m., intramuscular(ly); s.c., subcutaneous(ly); PBS, phosphate-buffered saline.

## Competing interests

Andres Salazar is the CEO of Oncovir, Inc, which provided us with poly-ICLC. However, all experimental data in the current study were evaluated completely independently by other authors who have no financial interests in the content of the current study.

## Authors' contributions

XZ carried out mouse surgery, BIL analyses and preparation of this manuscript. FN, MF and JE provided technical expertise in flow-cytometric evaluation of BILs. KS contributed in mAb-mediated *in vivo *blocking of CD49. JED prepared reagents and performed surgery on mice. WKF provided expertise for LFB staining of brain sections. WJS and PRW participated in peptide identification, the design of the study and critical review of data and the manuscript. As provided poly-ICLC as well as critical insights in the design of experiments based on his past experience with poly-ICLC clinical trials. HO conceived of the study, and participated in its design and coordination. All authors have read and approved the final manuscript.
